# Comparison of exposure of the feline radial diaphysis by the craniomedial and craniolateral surgical approaches for repair of antebrachial fractures

**DOI:** 10.1111/vsu.70001

**Published:** 2025-08-07

**Authors:** William Bower, Shane Guerin, Sorrel J. Langley‐Hobbs

**Affiliations:** ^1^ University of Bristol Bristol UK; ^2^ Veterinary Specialists Ireland Little Island Cork Ireland

## Abstract

**Objective:**

To report and compare two surgical approaches to the feline radial diaphysis and outline optimal techniques to maximize surgical exposure.

**Study design:**

Ex vivo experimental comparative study.

**Sample population:**

Left and right antebrachia of three feline cadavers.

**Methods:**

Six feline antebrachia were collected from cadavers submitted for research purposes. Each limb was sequentially dissected for both craniomedial and craniolateral surgical approaches totalling 12 surgical approaches, or 24 when including and excluding the supinator muscle. Following each approach, photographs were taken and inserted into an image measuring software, to obtain the area of surgical exposure. The datum was then analyzed to ascertain which approach afforded the greatest exposure and whether the difference was statistically significant.

**Results:**

The mean surface area for the craniolateral approach, including and excluding the supinator muscle, was 4.13 cm^2^ and 2.63 cm^2^, respectively. The mean surface area for the craniomedial approach, including and excluding the supinator muscle was 3.84 cm^2^ and 2.45 cm^2^, respectively. The difference observed between the two approaches was not statistically significant (*p* > .05).

**Conclusion:**

Surgical exposure achieved via the craniolateral and craniomedial approaches to the feline radial diaphysis is comparable.

**Clinical significance:**

This study validates the use of the craniolateral surgical approach to the feline radial diaphysis.

## 1 INTRODUCTION

Cats possess remarkable anatomical adaptations that enhance their agility and dexterity, setting them apart from dogs in both the treatment and management of fractures to their antebrachium. A key difference is that they exhibit a significantly greater degree of antebrachial pronation (40°–50°), and supination (90°–128°) compared to dogs, which possess almost half this range of motion.^1^ This enhanced range of motion is critical for activities such as climbing, grooming, and jumping. It is facilitated by the unique anatomical structure of the feline antebrachium, where the radius curves from a craniolateral position relative to the ulna proximally to a medial position distally.^2^ This was illustrated in a study by De Lima Dantas et al.,^3^ which standardized radial angles in cats, revealing that the average medial proximal radial angle (MPRA) was 70.97° while the average lateral distal radial angle (LDRA) was 91.72°. The aim of antebrachial fracture repair should be to maintain this degree of pronation and supination and restore function to near normal. The feline anatomy presents inherent challenges for the surgeon to achieve this, and correct positioning of the cat and visualization of surgical site is paramount to prevent complications.

Radial and ulnar fractures constitute between 3%–14% of all long bone fractures seen in cats.^2^, ^4^, ^5^ Complications following fracture repair at this site are frequently reported in the canine population.^6^, ^7^ One study found that radius and ulnar fractures are the site most associated with delayed or non‐union complications, compared with all other fracture locations in dogs.^6^ Particular attention is drawn to small and toy breed dogs less than 6 kg bodyweight, where radial and ulnar fractures are particularly prone to complications following surgical repair. This has been attributed to factors such as meager soft tissue coverage, poor vascularisation and limited contact between apposing fracture sites.^7^, ^8^, ^9^

Complication rates following antebrachial fracture repair are comparable between cats and toy breed dogs. A retrospective study described the outcome of 26 cats diagnosed with combined radial and ulnar fractures, treated surgically using a combination of techniques including external skeletal fixators (ESF), open reduction internal fixation (ORIF) and intramedullary pins.^10^ The rate of major complication in this population was 23%. This study defined a major complication to be one that required further intervention, either by surgical revision, amputation or external coaptation. Comparing these findings to ones found in a study of 47 dogs with a median weight range of 3 kg following radius and ulnar fracture repair.^9^ A similar rate of complication was found as 21% of dogs required surgical revision or amputation following fracture repair.

The feline antebrachium is complex; the paired bone system and the high degree of pronation and supination presents challenges to the surgeon in alignment, accurate reduction and rigid stabilization and restoration of function. As such, fixation of a singular bone (SBF), as seen in small breed dogs may provide insufficient support.^11^ Studies show dual bone fixation (DBF) with the addition of an intramedullary pin placed within the ulna of cats, provides lower complications rates than stabilization of the radius alone.^10^, ^12^ More recent research by Makar and others^13^ compared the outcome of radial and ulnar diaphyseal fractures treated with DBF or SBF. It found DBF to have a lower rate of major complication (7.7%), when compared with SBF (11.1%). However, long term outcomes of the two treatments were difficult to interpret due to the variable follow up.

The standard surgical approach to the canine radial diaphysis is craniomedial with proposed advantages including most direct access to the bone with a minimal amount of muscle dissection.^14^, ^15^ Two feline specific studies also described a craniomedial approach for open reduction and internal fixation (ORIF) of the radial diaphysis.^2^, ^16^ However, Johnson^17^ describes the craniolateral surgical approach to both the canine and feline antebrachium dissecting between the extensor carpi radialis muscle and the common digital extensor which is recommended if reduction of the ulna is needed in support of radial fixation. Additionally, for minimally invasive plate osteosynthesis (MIPO), the craniolateral approach has been recommended in cats, which differs from that seen in dogs due to the feline radius having a more pronounced lateral torsion.^18^ Positioning the cat on the operating table for a craniomedial surgical approach to the radial diaphysis, for applying the plate to the cranial surface of the bone can be challenging. The cat can either be positioned in dorsal recumbency with the affected leg tractioned caudally, or the cat is positioned in sternal recumbency with the leg tractioned in a cranial position. Positioning for a craniolateral surgical approach to the feline antebrachium is easier with the cat positioned in lateral recumbency and the affected leg can be naturally positioned without the need for traction.

To the authors' knowledge, comparisons in bone exposure between the craniomedial and craniolateral approach of the feline radius have not been made in the literature. Which approach affords the most exposure forms the basis of this study as greater visualization may lead to increased ease and accuracy in fracture reduction and enable the cat to be positioned in a lateral position which is easier for the surgeon without an assistant.

## 2 MATERIALS AND METHODS

Feline antebrachia were collected from cats euthanised for reasons unrelated to the study. Three feline cadavers were donated by clients that signed a consent form disclosing that they be used for educational and research purposes. The antebrachia were surplus to a student dissection study. The cadavers were fresh frozen in −80°C and thawed 12 h prior to the study. The specimens were kept in moist conditions.

Power analysis to obtain a sample size was not conducted in this study. The absence of prior information and research limits the availability to produce an accurate effect size. Additionally, the use of cadaveric models as seen in this study, presents inherent challenges in their availability, as centers rely on them being donated for research purposes. Therefore, a total of 12 surgical approaches were conducted from six feline thoracic limbs, creating six data points per approach. The cadavers were examined prior to the procedure, assessing for any deformities or fractures that could affect the validity of the results. The whole cadavers were weighed prior to starting the study and the three cadavers were identified as A, B and C. Sequential dissections were then performed on each of the cadavers.

The antebrachia was clipped of hair extending from the carpus to the elbow exposing the proposed surgical site. For each limb, a craniomedial and craniolateral approach were used in an alternate manner. Two small pairs of Gelpi retractors were placed at the most proximal and distal end of the incision, as would be utilized in clinical cases, and a small Hohmann retractor placed centrally retracting the extensor muscles of the limb. The Gelpi retractors were opened at the same setting in all cases to ensure uniformity throughout the data collection.

**FIGURE 1:**
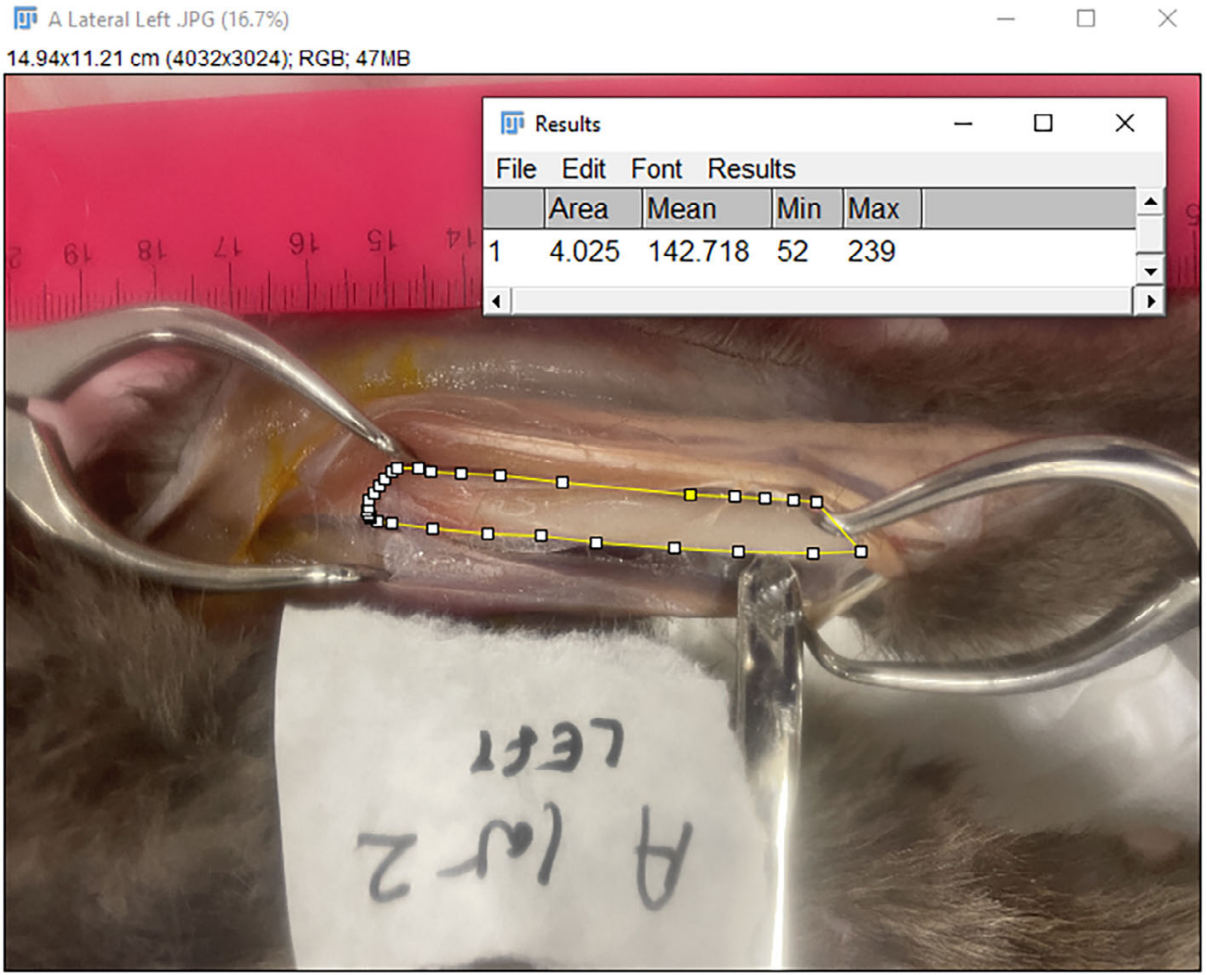
Cadaver A, left limb craniolateral approach including the supinator muscle.

**FIGURE 2:**
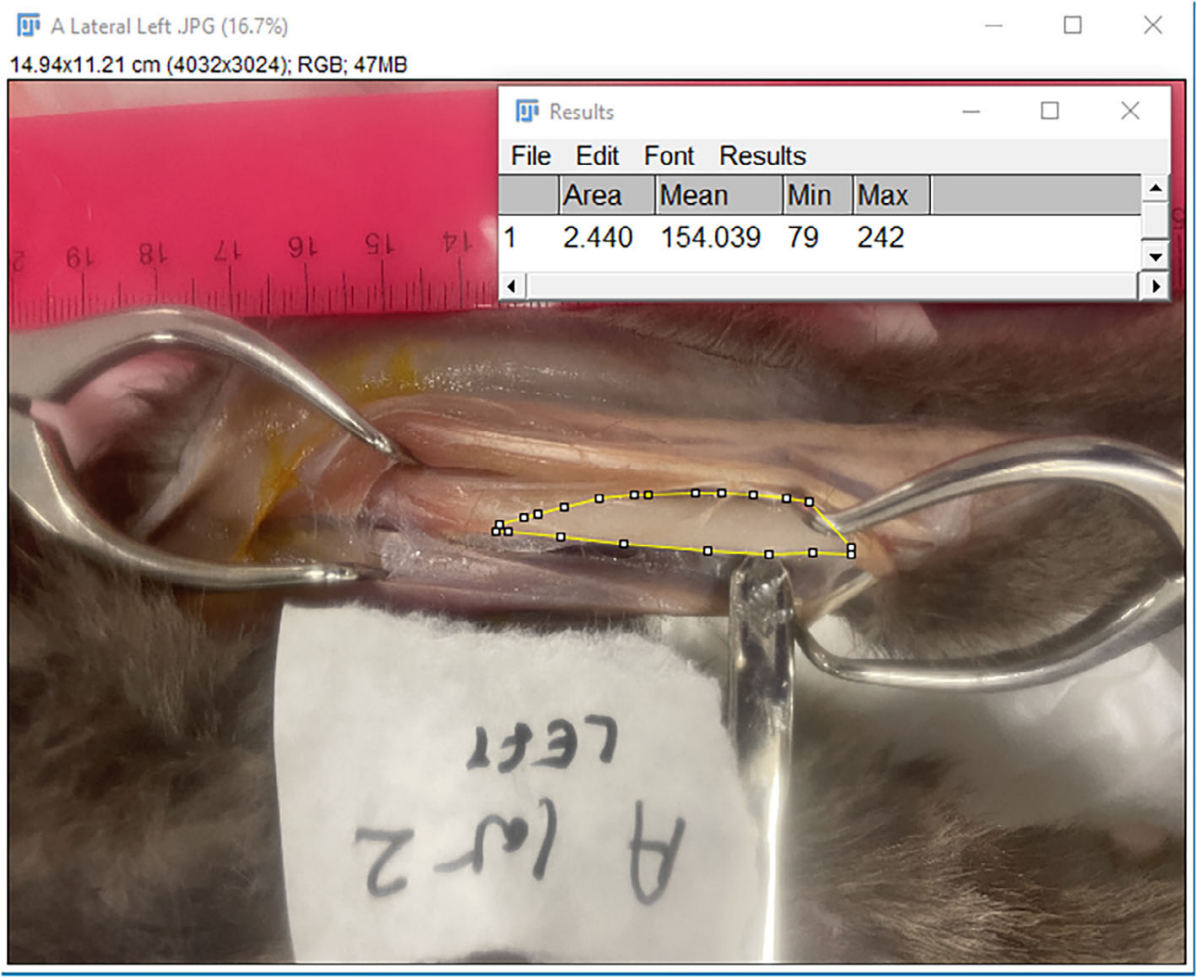
Cadaver A, left limb craniolateral approach excluding the supinator muscle.

Following each surgical approach a photograph was taken with a digital camera from the surgeon's vantage point of the exposed bone. The images were then inserted into ImageJ for further analysis, which allowed for the quantification of the area of exposed radius on a two‐dimensional calibrated digital image. A polygon selection tool was used to trace an outline of the exposed radius. Two measurements were taken for each approach including and excluding the supinator muscle giving a total of 24 surgical approaches (Figures 1 and 2).

Statistical analysis was performed using SPSS (IBM) comparing the craniomedial and craniolateral approach using the values of the surface area of the six antebrachia. A linear mixed model was used to test for significance between the two approaches. A threshold for significance was established at *p* ≤ .05.

To mitigate the confounding variables of this study, each of the cadavers were frozen in the same conditions and thawed at the same time, as not to induce variability in the tissue stiffness or pliability. A single board‐certified small animal surgeon made each of the surgical approaches, which were conducted in an alternate randomized order. The incision extended from the elbow to the carpus to avoid being limiting factor. Ninety millimeter Gelpi retractors opened with a jaw gap of 30 mm created consistent tension across each of the surgical sites. The images were taken by the same camera and operator with lighting and conditions kept the same. A single person analyzed the images using ImageJ as not induce variability in the measurements.

## 3 RESULTS

None of the specimens demonstrated any visual or physical evidence of injury of the antebrachium, nor was there evidence of previous surgery.

The mean surface area for the craniomedial and craniolateral approaches including the supinator were 3.84 cm^2^ and 4.13 cm^2^, respectively (Table 1). For the approach excluding the supinator muscle the mean craniolateral approach was 2.63 cm^2^ and the craniomedial 2.45 cm^2^ (Table 2).

**TABLE 1:** Measured area of surgical approach including the supinator muscle.

Surface area of surgical approach including supinator muscle				
Cadaver	Weight (kg)	Foreleg	Surface area (cm^2^)	
			Craniomedial	Craniolateral
A	3.175	Left	4.19	4.03
		Right	3.96	2.90
B	2.75	Left	3.35	5.15
		Right	5.66	4.65
C	2.6	Left	2.96	3.82
		Right	2.88	4.24
		Mean	3.84	4.13

**TABLE 2:** Measured area of surgical approach excluding the supinator muscle.

Surface area of surgical approach excluding supinator muscle				
Cadaver	Weight (kg)	Foreleg	Surface area (CM2)	
			Craniomedial	Craniolateral
A	3.175	Left	2.65	2.44
		Right	2.45	1.64
B	2.75	Left	2.66	3.46
		Right	3.29	3.30
C	2.6	Left	1.77	2.28
		Right	1.91	2.65
		Mean	2.45	2.63

A linear mixed model was used to detect whether the difference between the two approaches was statistically significant. In the model: approach, limb and weight were included as fixed effects. Cadaver ID (A, B and C) was selected as a random effect, to account for the repeated measures within the individuals.

In the model including the supinator muscle, none of the fixed effects reached statistical significance: approach (*F* = 0.258, *p* = 0.625), limb (*F* = 0.050, *p* = 0.282) and weight (*F* = 0.005, *p* = .943). The marginal *R* ^2^ was 0.038, outlining that the fixed effects explained only 3.8% of the variance in surface area.

In the model excluding the supinator, comparable results were found with no significant effects: approach (*F* = 0.192, *p* = .673), limb (*F* = 0.000, *p* = .993) and weight (*F* = 0.108, *p* = .751). The marginal *R* ^2^ was 0.0036.

Comparisons were made between the estimated marginal means for each approach with the cadaver weight controlled at a fixed value. The model standardized weight at 2.8421 kg which estimated the craniolateral and craniomedial approach to give a surface area of 4.132 cm^3^ and 3.833 cm^3^, respectively when including the supinator. The difference between the estimated means was 0.299 cm^3^. Similarly, when evaluating the predicted means excluding the supinator. The craniolateral and craniomedial approach gave an estimated surface area of 2.628 cm^3^ and 2.455 cm^3^, respectively with a difference of 0.173 cm^3^.

## 4 DISCUSSION

This study aimed to compare the surface area of exposure of the feline radius using either craniomedial or craniolateral surgical approaches, with and without elevation of the supinator muscle, in a controlled cadaveric model. No statistically significant difference was observed between the two approaches. When comparing the estimated marginal means, the surface area following a craniolateral approach appeared to be larger than craniomedial in both datasets. The difference was small, with confidence intervals that overlapped, validating that the difference would not be significant if weight was fixed across all cadavers. Despite this, the datum demonstrates that the craniolateral approach provides comparable exposure to the traditional craniomedial approach, whilst offering practical advantages. Specifically, the craniolateral technique allows for a relaxed positioning of the cat without requiring traction or a theater assistant to hold the limb during surgery and it enables dual bone fixation.

The use of cadaveric models, although necessary for this study, introduces limitations, particularly the small sample size, as specimens are often limited to those available for research. To maximize the datum available, both left and right limbs were used, which created a total of 12 surgical approaches with six data points per approach. This sample size is comparable to other studies which focus on human cadavers. Wu et al.^19^ evaluated the surgical exposure of four different surgical approaches to the distal humerus, as with this current study, each approach had six data points. Other studies also use a comparable methodology with a similar sample size, which provides further validation of this current study design.^20^, ^21^ A linear mixed model was selected for the analysis of the data as it has been described to provide adequate statistical power when testing as few as five units, if the question relates to the fixed effects, which, in the case of this paper, was surgical approach.^22^ The mixed model also allows for the inclusion of random effects—such as cadaver ID—allowing for a more accurate generalization of the variability within a population, instead of assuming that all subjects respond identically.^23^

Radiographic studies which describe the radius and ulna in both dogs and cats mainly include images composed exclusively of the canine antebrachia.^24^ These studies therefore do not accurately represent the degree of rotation observed in the feline antebrachii during surgical exposure; making it more challenging to plan which positioning and approach is most suitable for the case. Modeled CT scans of the feline antebrachium are particularly useful at highlighting this, which may be more practical in surgical planning, as they provide a more accurate representation of the feline anatomy.^25^ The inherent rotation of the radius and ulna seen in cats makes accurate fracture reduction challenging. Patient positioning plays a crucial role in the success of fracture reduction, especially in cases involving comminution, where the loss of intrinsic support complicates reduction efforts. A retrospective study in cats found that fractures with severe comminution of the radius and/or ulna can have surgical revision rates of up to 60% (*n* = 5). The three cases which required surgical revision were treated initially with external skeletal fixators (ESF). The one case with severe comminution treated with DBF healed with no major complication.^10^ In the same study, comparisons were made between SBF and DBF which found the surgical revision rate was lower (12.5%) in DBF compared with SBF (27%). Other studies have validated the use of DBF in the surgical repair of the feline antebrachium,^12^, ^26^ suggesting that DBF mitigates the increased axial and rotational forces seen between the radius and ulna, reducing complications and the need for surgical revision. Additionally, DBF provides the practical advantage of allowing initial stabilization of the ulna to facilitate a more precise reduction of the radius. In such cases where DBF is selected, the craniolateral approach would be preferable to the craniomedial as it facilitates stabilization of both the ulna and radius without the need for repositioning or making two separate skin incisions or surgical approaches.

If the craniomedial approach is selected, caution must be taken when elevating the pronator and supinator muscles to protect the integrity of the median and radial nerves, which are vulnerable to damage due to their proximity to these structures.^17^ The craniolateral approach mitigates this risk as caudal retraction of the lateral digital extensor exposes most of the lateral shaft of the radius.

The main limitation of this study was the use of cadaveric models, which, while practical, do not fully replicate clinical conditions. In a live surgical setting, factors such as muscle tension, mobile fractures and bleeding can complicate visualization, affecting both approaches. Additionally, subtle differences in the mechanical interactions between the instrumentation and tissues may affect the surgical exposure. Another limitation was the small variation in weight among the cadavers used, which only ranged from 2.6 kg to 3.1 kg. To address this, future studies may want to use wider weight ranges allowing more direct comparisons to be made. Despite this, the impact on the overall findings is likely minimal.

## 5 CONCLUSION

To conclude, the craniolateral surgical approach for feline radial fractures provides comparable bone exposure when compared to the craniomedial approach. As such this technique can provide a valuable addition to the armamentarium of the feline orthopedic surgeon and is beneficial in cases that require DBF or ones with a solo surgeon.

## AUTHOR CONTRIBUTIONS

Bower W, BSc, BVSc, MRCVS: Interpreted and analyzed the data for statistical significance, drafted the body of text, researched around the topic and revised the manuscript. Guerin S, MVB, MACVSc, CertSAO, DVCSc, DECVS, MRCVS: Contributed to the concept and design of the study, revised drafts of text. Langley‐Hobbs SJ, MA, BVetMed, DSAS(O), DipECVS, FHEA, FRCVS: Responsible for the acquisition of data, concept and design of study and revising important information critically. All authors critically reviewed the manuscript and validate the final copy.

## CONFLICT OF INTEREST STATEMENT

This study had no grant or other financial support, nor is there any other conflict of interest related to a company or product.
